# Disentangling organizational levers and economic benefits in transitional care programs: a systematic review and configurational analysis

**DOI:** 10.1186/s12913-023-10461-3

**Published:** 2024-01-09

**Authors:** Stefano Landi, Maria Martina Panella, Chiara Leardini

**Affiliations:** 1 Department of Management, Università di Verona, Via Cantarane, 24, 37129 Verona, Italy; 2 IRCCS- Azienda ospedaliera universitaria Bologna, Policlinico di S.Orsola-Malpighi, Via Pietro Albertoni, 15, Bologna, Italy

**Keywords:** Transitional care program, Components, Healthcare management, Readmissions reduction, Hospital re-admissions, Economic evaluation, System

## Abstract

**Background:**

Promoting safe and efficient transitions of care is critical to reducing readmission rates and associated costs and improving the quality of patient care. A growing body of literature suggests that transitional care (TC) programs are effective in improving quality of life and reducing unplanned readmissions for several patient groups. TC programs are highly complex and multidimensional, requiring evidence on how specific practices and system characteristics influence their effectiveness in patient care, readmission reduction and costs.

**Methods:**

Using a systematic review and a configurational approach, the study examines the role played by system characteristics (size, ownership, professional skills, technology used), the organizational components implemented, analyzing their combinations, and the potential economic impact of TC programs.

**Results:**

The more organizational components are implemented, the greater the likelihood that a TC program will be successful in reducing readmission rates. Not all components have the same effect. The results show that certain components, ‘post-discharge symptom monitoring and management’ and ‘discharge planning’, are necessary but not sufficient to achieve the outcome. The results indicate the existence of two different combinations of components that can be considered sufficient for the reduction of readmissions. Furthermore, while system characteristics are underexplored, the study shows different ways of incorporating the skill mix of professionals and their mode of coordination in TC programs. Four organizational models emerge: the health-based monocentric, the social-based monocentric, the multidisciplinary team and the mono-specialist team. The economic impact of the programs is generally positive. Despite an increase in patient management costs, there is an overall reduction in all post-intervention costs, particularly those related to readmissions.

**Conclusions:**

The results underline the importance of examining in depth the role of system characteristics and organizational factors in facilitating the creation of a successful TC program. The work gives preliminary insights into how to systematize organizational practices and different coordination modes for facilitating decision-makers’ choices in TC implementation. While there is evidence that TC programs also have economic benefits, the quality of economic evaluations is relatively low and needs further study.

**Supplementary Information:**

The online version contains supplementary material available at 10.1186/s12913-023-10461-3.

## Introduction

Healthcare systems around the world are paying programmatic attention to reducing hospital readmission rates. They are considered an indicator of quality of care [1] and a common burden on healthcare systems, with reduced readmissions potentially reducing costs and improving quality of care. While some readmissions are unavoidable, due to the natural course of disease or worsening of chronic conditions, readmissions also occur because of suboptimal quality of care or poor communication between different care settings. Hospital readmissions have long been of interest to researchers, and even more so in the last 15 years, when specific policies and strategies have been focused on reducing readmission rates [2]. For example, several countries have introduced both financial or non-financial incentives to reduce readmissions, which may take the form of bonuses or penalties such as a reduction in reimbursement to the hospital or the introduction of publication and transparency of readmission rates [3]. Along with these policies, there has been an increasing focus on the components that occur at the point of transition from hospital to home, as this is a vulnerable period of discontinuity and an area of emerging costs, even more so today and in the future given an ageing population with more long-term needs [4].

Promoting a safe and efficient transition of care is critical to reducing readmission rates and associated costs and ultimately improving the quality of patient care. It is therefore important to reduce the number of avoidable hospital admissions and to implement integrated discharge programs that can ensure continuity of care after hospital discharge [5–6].

The concept of continuity of care emerges in response to the implementation of hospital discharge planning; although this concept is present in the literature, it is “more often assumed than defined” [7].

**Fig. 1:**
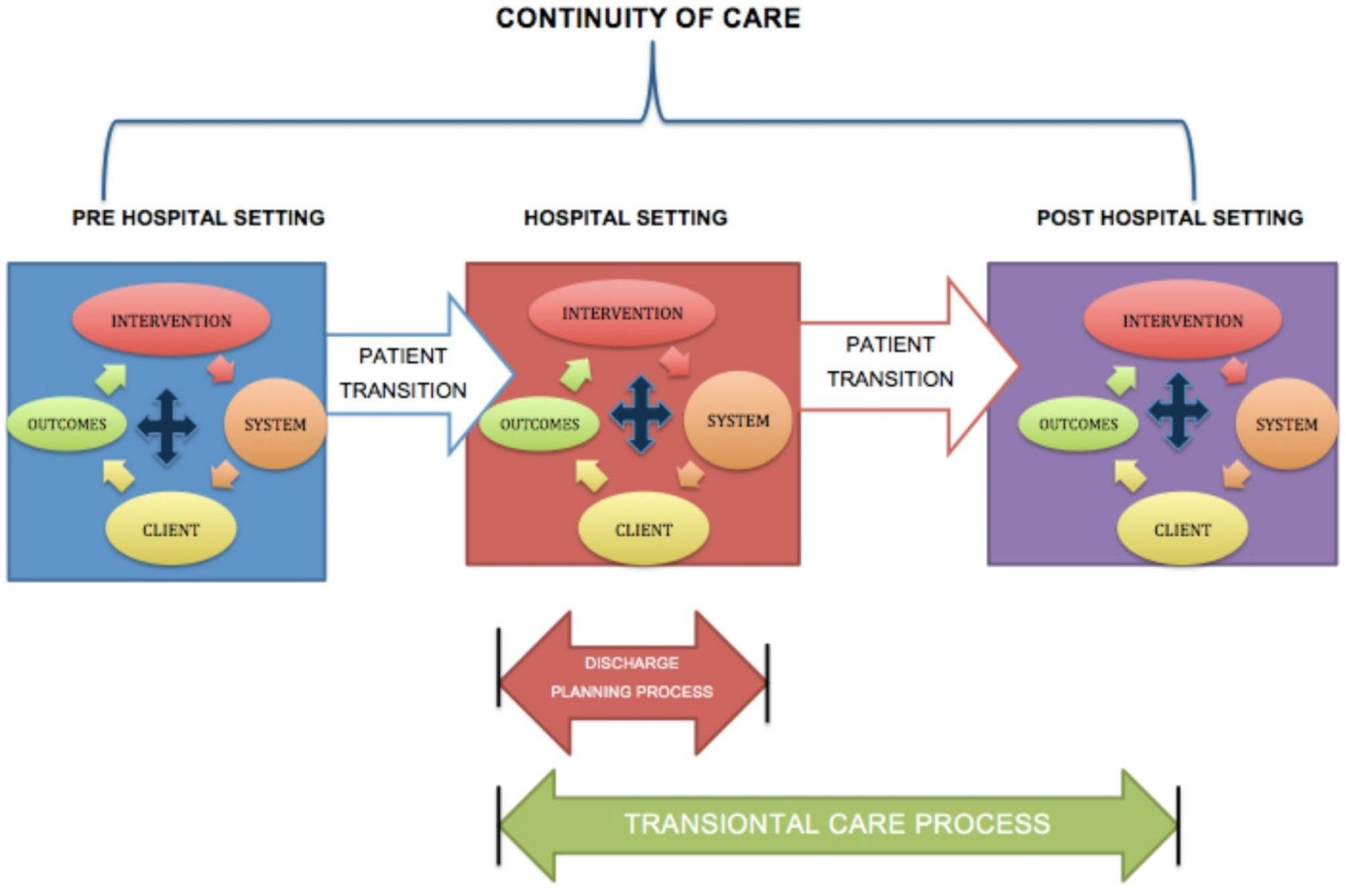
Continuity of care vs. transitional care process. Adapted from Holland et al. 2007

A transitional care intervention is a process of care under the umbrella of continuity of care [8] together with discharge planning interventions. Transitional care began to be discussed in the 1980s and is a process of care that consists of support services rather than follow-up activities that begin before hospital discharge in the post-hospital setting [6]. It can be defined as “a broad range of time-limited services designed to ensure continuity of care, prevent avoidable poor outcomes in vulnerable populations, and promote the safe and timely transfer of patients from one level of care to another or from one type of setting to another“ [8]. The definition of transitional care isn’t specific about the events that mark the beginning and end of transitional care, but it is typically outlined in terms of support services, follow-up and other activities that range from pre-hospital discharge to post-hospital settings. This is a very different concept from pure discharge planning, which is limited to a hospital stay and a discharge event. These two concepts can be identified graphically as shown in Fig. 1.

A growing body of literature suggests that transitional care (TC) programs are effective in improving quality of life, reducing mortality and unplanned readmissions for several patient groups, and have the potential to generate cost savings by reducing readmissions and improving outpatient management [9–12].

Several papers and systematic reviews have shown that, compared with usual care, transitional care services for patients discharged from hospitals reduce overall health system costs and have an impact on hospital readmission rates, quality of life and cost-effectiveness [13–15]. The effects of transitional care on quality of life and rehospitalization may vary in effectiveness depending on the components or intensity [8, 16].

TC programs are highly complex and multidimensional. First, they are characterized by multiple components that can be combined in different configurations depending on the context and available resources. TC programs may share certain components but may also include distinctive elements. The choice of components and their combination is important in designing an effective program. Second, the system in which the program is developed can significantly impact its success. A TC program produces its clinical results through organizational factors and the characteristics of the patient to whom the component is delivered [17–18].

Emerging research has begun to explore the influence of specific practices on the effectiveness of transitional care programs [19] Despite these relevant contributions, there is a lack of a comprehensive approach that combines organizational practices with clinical evidence to support those wishing to implement a TC program. A decision-maker wishing to implement a TC program is faced with several open questions, such as: what type (and number) of components should be implemented? Which categories of professionals should be involved and how should they be coordinated? Which patient profiles will benefit most from the component? Under what circumstances is TC cost-effective?

These are important gaps, as there are strong pressures on health systems to improve the choice of value for money components from multiple directions and the implementation of a TC program requires several organizational decisions. Many studies have reported results on individual components of a TC program [16], but there is a lack of systematization of the different combinations of organizational practices that can help decision-makers choose the most appropriate model for their specific case. Furthermore, skills and social relations can be crucial to the realization of TC benefits. Thus, the choice of skills and the type of professional involvement need to be studied in addition to the type of component.

The present paper aims to answer these questions in a systematic way for the first time. Using a systematic review and a configurational approach, it examines the role of system characteristics (size, ownership, professional skills, technology used), the components implemented (TC components) and the choice of target patient in creating a successful TC program. It also assesses the potential economic benefits in terms of cost savings or cost-effectiveness of TC programs from the perspectives of health organization, third payer and society.

## Method

### Design and setting

This study is a systematic review of TC programs that focus on organizational levers and economic domains. According to Donabedian (1966), the program produces its clinical outcomes by always acting through organizational factors and characteristics of the client to whom the component is delivered [17]. The main organizational factors analyzed are the system characteristics and the components implemented.

The *system characteristics* consider the structural variables of organized agencies (such as a hospital or provider network), including elements such as size, ownership, professional skill mix involved, type of technology used, and ancillary care service across the care continuum, which are able to affect processes and produce desirable or undesirable outcomes. Transitional care (TC) systems can also have two primary settings: the hospital from which the patient is discharged and community-based settings such as nursing homes, family homes, or the household where the patient arrives.

The *components* refer to the direct or indirect clinical processes and related activities or components through which the program is delivered. To analyze this level accurately, we build on the Ideal Transition in Care framework, which maps the different possible activities of TC and positions them across the continuum of care from hospital to home [16]. The framework proposes 10 domains (see Table 1). Success in reducing readmissions is associated with the number of components included in the TC component [16]. Furthermore, using logistic regression, they found that not all domains were associated with the same effect on reducing readmissions. The individual domains most associated with reducing readmissions were monitoring and managing symptoms after discharge, using social and community support, and educating patients to promote self-management.

**Table 1:** Details of the ITC dimensions implemented for each study analyzed

Dimension	Complete Communication of Information	Availability, Timeliness, Clarity, and Organization of Information	Medication Safety	Educating Patients, Promoting Self-Management	Monitoring and Managing Symptoms after Discharge	Enlisting Help of Social And Community Supports	Advance Care Planning	Coordinating Care Among Team Members	Discharge Planning	Follow-Up with Outpatient Providers		
Dimension description	Focuses on the content of the information delivered to the receiving clinician	Highlights if/when this information is received by the receiving clinician, and how it is optimally presented to maximize utility	Medication reconciliation across the continuum of care	Education to patients and caregivers, using principles of health literacy, teach-back, and encouraging self-advocacy	Multi-modality interventions (telehealth, calls, visits in clinic and/or home), and a responsible clinician to respond to concerns	Adequate assessment of home environment and support and implementing help if needed	Establish health-care proxy and goals of care	Share medical records, communicate with all team members, optimize continuity of providers, formalize handoffs	Emphasize identifying patient needs prior to discharge, implementing interventions prior to discharge	Follow-up with the right provider(s), appropriate time frame, preparation for visit		
Author	Year											*N* intervention implemented per TC
Moore et al.	2017	x	x	x		x				x		5/10
Hall et al.	2014	x		x	x	x			x	x		6/10
Wong et al.	2012			x	x	x	x			x		5/10
Harrison et al.	2014	x		x		x			x			4/10
Wong et al.	2015	x		x		x	x	x	x	x	x	8/10
Stranges et al.	2015	x	x	x		x			x	x	x	7/10
Anderson	2005	x	x	x	x	x			x	x	x	8/10
Courtney et al.	2009	x			x	x	x	x		x	x	7/10
Galbraith et al.	2017				x	x				x	x	4/10
Jackson et al.	2013			x	x	x				x	x	5/10
Watkins et al.	2012			x	x	x	x	x		x	x	7/10
Stauffer et al.	2011	x	x	x	x	x	x	x	x	x	x	10/10
Naylor et al.	2004	x	x	x	x	x	x	x	x	x	x	10/10
Jingyi et al.	2018	x	x	x	x	x	x		x	x	x	9/10
Jackson et al.	2016					x	x					2/10
Russel et al.	2011	x	x	x	x	x	x	x	x	x	x	10/10
Saleh et al.	2012			x	x	x	x	x	x	x	x	8/10
Kam et al.	2015			x		x	x			x	x	5/10
Zhang et al.	2017			x	x	x	x		x	x	x	7/10
Dhillon et al.	2017					x				x	x	3/10
Simpson	2014			x	x	x	x	x	x	x	x	8/10
Coleman et al.	2006	x	x	x		x				x	x	6/10
Naylor et al.	1999	x	x		x	x	x	x	x	x	x	9/10
Naylor et al.	1994	x	x		x	x	x	x	x	x	x	9/10
Rich et al.	1995				x	x	x			x	x	5/10
Balaban et al.	2015			x		x	x			x	x	5/10
Graves et al.	2009	x	x		x	x				x	x	6/10
Kwok et al.	2008			x		x				x	x	4/10
Wong et al.	2008				x			x		x	x	4/10
Gardner et al.	2014	x	x		x	x			x	x	x	7/10
Lee	2017					x				x	x	3/10
Baley et al.	2019	x	x	x	x	x	x			x	x	7/10
Candelario et al.	2018			x	x	x	x		x	x	x	7/10
Kripalani et al.	2019			x	x	x	x		x	x	x	7/10
Lee et al.	2019			x	x	x					x	4/10
Rahim et al.	2018			x	x	x				x	x	5/10
Taylor et al.	2019			x	x	x				x	x	5/10
Xiang et al.	2018			x	x	x	x	x	x	x	x	8/10
Total		17/38	13/38	27/38	27/38	37/38	21/38	12/38	18/38	35/38	33/38	

To confirm and enrich previous findings, this paper has adopted a configurational approach to analysis, which allows variables to be treated as combining rather than competing to produce an outcome [16, 20]. TC programs are complex constructs with multiple dimensions that often, but not necessarily, interact with each other. A TC program may use multiple and diverse implementation strategies with different combinations of domains. Therefore, the idea that some specific domains have an independent net effect on the likelihood of success of a TC program, independent of the use of other domains, is challenged by the causal complexity of the outcome.

Several clinical trials use the readmission rate as the primary outcome to evaluate the effectiveness of TC, and health systems around the world are paying programmatic attention to reducing hospital readmission rates [1]. Indeed, fewer unplanned readmissions have the potential to reduce costs and improve the quality of care. We conduct the analysis using qualitative comparative analysis (QCA). This is a case-oriented method for studying complex phenomena that originated in the comparative social sciences and has been proposed as a useful method for synthesizing evidence in systematic reviews of multidimensional health components [21–22]. Based on Boolean algebra, QCA allows asymmetric and equifinal solutions for a given outcome, identifying complex (i.e. non-linear, non-additive) causal patterns that variable-oriented methods may miss. This methodology also allows us to find necessary or sufficient areas to be implemented to create a successful TC program.

Finally, adopting the TC program also raises the issue of assessing its economic impact and cost-effectiveness. The Drummond 10-point scale [23] was used to assess the quality of the studies and the main findings.

### Data extraction

**Fig. 2:**
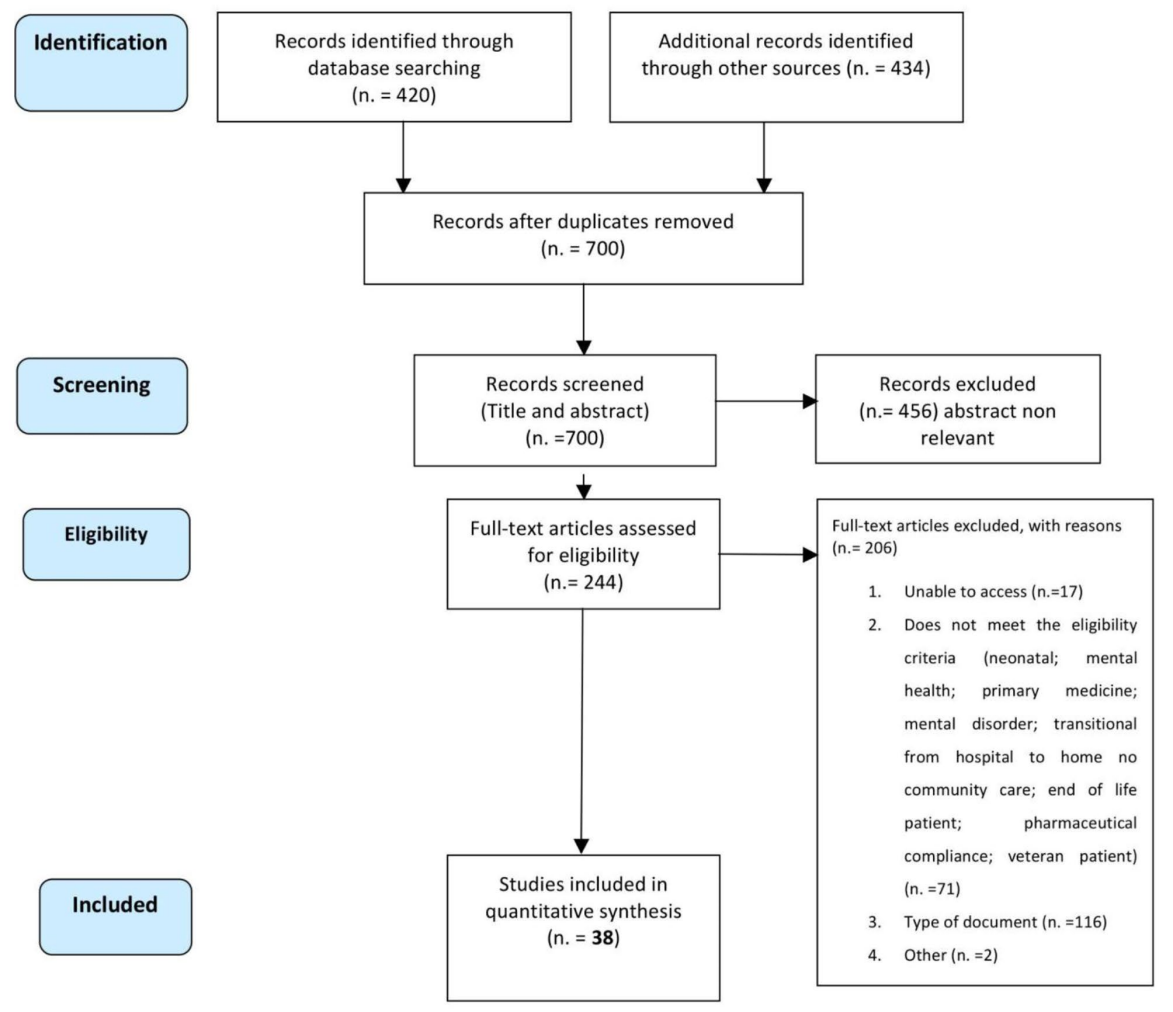
PRISMA diagram—Information flow chart of the different steps of the systematic review

The article selection process follows the phases of a systematic review and is visually conceptualized in the Prisma diagram in Fig. 2.

Studies examining the effects of TC that were published in the English language were identified through electronic bibliographic databases and manual research from 1994 through January 2021. The databases consulted were PubMed/MEDLINE, Scopus, Web of Science, and CINAHL using the same algorithm (“transitional care” OR “transition care” OR “care transition program” OR “transitional discharge model”) AND (“hospital* readmission” OR “hospital* re-admission”) AND cost*.

The reference population was the entire population, except for oncology and end-of-life patients, patients with mental disorders, neonatal and pediatric patients, and veterans. The intervention is the implementation of transitional care models compared to standard care. The outcome measures of interest are readmissions and costs. The study designs allowed are RCTs and observational studies. Therefore, studies were included for full-text review if, after reading the abstract, they met the inclusion criteria reported below: (i) the primary objective was to evaluate the efficacy of a transitional care intervention to reduce readmission rate during the discharge from the hospital to home; (ii) analyses primary empirical data. Furthermore, we stated the following exclusion criteria: (i) neonatal and pediatric patients; (ii) primary medicine; ii) oncologic and end of life patients; (iii) mental disorder; (iv) veteran patients; (v) pharmaceutical compliance; (vi) costing studies describing costs only; (vii) discussion papers, letters or commentaries.

Using this approach, 854 studies were initially located, and their titles were examined. Of these 854 records, after the removal of duplicates, 700 abstracts were read by two reviewers. After screening the abstracts, 244 entire articles were examined further. Of these, 206 articles did not meet the eligibility criteria. Finally, 38 articles remained as fully meeting the eligibility criteria. CL and MM developed the search strategy. MM conducted the initial screening of titles and abstracts. Full-text screening was performed by all authors. Authors held regular meetings to discuss discrepancies. Disagreements were resolved through discussion and consensus. Data extraction was conducted by MM and SL independently. Any discrepancies were resolved through consultation with CL.

In Supplementary Table 1 there is a table summarizing the main results of the study included [11, 24–54].

## Results

### Main results

The increase in interest in TC programs is evident by the fact that more than half of the studies were published after 2014 (Supplementary Image 1). The majority of studies were implemented in the United States and, in particular, after 2010, the year in which the *Patient Protection and Affordable Care Act* was signed. The remaining studies on TC were conducted in China (six studies) and Australia (two studies), and Iran (one study), while studies in the European context appear to be absent.

The main outcomes achieved by TC programs were shorter hospital stays, significantly longer time to first readmission, and reductions in readmission rates at 30, 60, and 180 days. The review confirms a positive association between TC programs and the two main outcomes: readmission rates and costs. Specifically, 26 out of 38 studies reported a positive association between TC and a reduction in readmission rates (30 days). Twenty-one studies included costs in their analysis, and all but two showed a reduction in healthcare costs for the TC group.

### System characteristics

In all the papers analyzed, the system component was examined in a limited way. Some studies reported the typology of the structure (e.g. regional hospital, academic hospital), size (number of beds), ownership (e.g. for-profit, non-profit, American safety net hospital or non-Medicaid recipients or alliances), but none explicitly highlighted the link between these characteristics of the system and the success of the component.

More attention was paid to the presentation of the skill mix of the professionals involved: 25 out of 38 reported in detail on the skills and the different roles that were played in the TC programs. The most common professionals were advanced practice nurses (nurses with superior competence and experience) [24], nurse care managers (those responsible for supporting patients), community nurses (community health nurses with a focus on communities) (i.e. [25–27] and other professionals with high social skills, such as social workers, patient navigators (those responsible for helping patients with the logistics of navigating a complicated system to optimize post-discharge care) [28], transition navigators, transition coaches (whose role is to “encourage the patient and carer to take a more active role in care transitions, provide continuity across settings and ensure that the patient’s needs are met regardless of the care setting”) [29]. Four organizational models seem to emerge, depending on the professionals involved and the mode of coordination between them.

Two are monocentric, with one professional in charge of coordinating the whole TC process. Depending on the skill mix of the professional in charge of the process, this model can have two different declinations. In one model, the professional in charge has high medical/health skills or is a ‘master prepared nurse’ [24], as is the case with advanced practice nurses, nurse care managers and community nurses, whereas in the second model, the individual in charge has high social skills, as is the case with social workers, patient navigators, transition navigators or transition coaches [37–39].

The *health-based, monocentric* model relies on the medical competence of the transition manager. For example, it is reported the importance of the advanced practice nurse ability to educate patients and families about symptoms, demonstrate appropriate self-management strategies, and improve communication between patients and healthcare providers to ensure patient adherence to the treatment plan led to the success of a TC program [11]. In this model, it is essential to have a person with high medical skills in the specific area as well as patient management skills. For example, nurses were selected from among those with several years of experience in cardiac surgery and practice in a structured communicative environment that included several meetings with surgeons and with patients and their families [25].

The *social-based monocentric* model is based on the social and cultural competence of the person charged with transitioning. For example, the patient navigators are ‘uniquely effective’ after discharge because they are able to engage vulnerable patients by connecting them to a network of community resources - as they are ‘lay people from the community who share the patient’s language and culture’ [37]. In the same vein, some authors identified the transition navigator-a social worker- as a resource capable of understanding and overcoming patients’ various environmental barriers [38]. They are able to assist patients in keeping in touch with the health system, outpatient providers, social services, and community resources by reporting on the patients’ social and health needs and ensuring continuity of care.

In concrete terms, those in charge of TC must direct patients to the specialists or services they need to integrate various treatments by coordinating them. Generally, they start identifying suitable patients for the component to participate in the TC program as soon as possible after admission, involving them in advance to anticipate their needs at discharge and conducting daily transition meetings with any clinical specialists to coordinate the provision of components tailored to their needs. Then they usually perform a structured needs assessment by identifying modifiable environmental barriers to facilitate early discharge planning and prioritization of services. After discharge, they perform a structured phone call to assess symptoms that require additional management, review medications, strengthen education, confirm follow-up appointments, and resolve pending items (e.g., test results, services domiciliary). Once this is done, they examine any new or worsening symptoms during phone calls, review medications, provide advance guidance, and, if necessary, monitor the patient through further and more frequent phone calls.

Then there are two polycentric models in which the responsibility for the TC process is shared among several professionals in a polycentric management. These can be developed in two different ways: (i) by a multidisciplinary team (i.e., [40–43, 26] or (ii) by a mono specialist team (i.e., two nurses, two patient navigators, two social workers) (i.e., [34, 44–45]. To address the multidimensional needs of the frail population (medical, social, psychological, functional), the multidisciplinary team is usually composed of professionals such as a medical specialist, a nurse, a social worker, and a pharmacist. Each person has different roles; for example, the physician conducts the multidimensional assessment, the pharmacist is responsible for identifying and adhering to medication therapy [28], and social workers usually assess the patient’s living situation and activities of daily living and home care with carers. The components of the team integrate and coordinate with each other mainly at two specific moments: a kick-off meeting for a multidimensional patient assessment and periodic monitoring meetings to discuss the different cases. A mono specialist team is based on the participation of specific professionals, and the same component steps described above for the monocentric model are distributed among the team.

### Component

Only three out of 38 studies implemented all dimensions of the ITC framework [11, 24, 30], but it is interesting to note that 30 out of 38 studies implemented at least five of the 10 dimensions.

The most implemented dimension was “Monitoring and Managing Symptoms after Discharge” followed by “Discharge Planning” and “Follow-up with Outpatient Providers.” It is interesting to observe that the least importance was attributed to “Advance Care Planning,” followed by “Complete Communication of Information” and “Availability, Timeliness, Clarity, and Organization of Information.”

The types and number of components were compared with the ability of the TC program to reduce readmission. The study was considered successful when the readmission rate at 30 and 60 days was statistically (*p*-value < 0.001) decreased. There is an association between the number of components implemented and the probability of success. The studies showing a statistically significant reduction in readmission had an average number of components of 7.5 versus 4.5 (*p* < 0.000).

Using QCA, we found different possible combinations (called configurations) of components leading to readmission reduction. First, we ran a necessary condition analysis, considering consistency the criterion for a condition to be necessary for an outcome and setting the threshold at 0.9. As Table 2 shows, “Monitoring and Managing Symptoms after Discharge” and “Discharge Planning” are necessary conditions for a TC program to be successful. This means that the result does not occur in their absence; therefore, they are important enough to be a necessary part of the program, regardless of the component combinations present. Next, sufficient configurations were obtained. The results indicate that two different combinations of components lead TC programs to reduce readmissions (see Supplementary Table 2 for the extensive Qca analysis).

**Table 2:** Configurations of interventions leading to an effective TC program in terms of readmission at 30 and 60 days (see complete analysis in supplementary material)

	Necessary interventions	Intervention 1	Intervention 2
Conf. 1	Monitoring and Managing Symptoms after Discharge Discharge Planning	Coordinating Care Among Team Members	
Conf. 2		Educating Patients, Promoting Self-Management	Enlisting Help of Social and Community Supports

The first combination, together with the necessary components, includes “Coordinating Care Among Team Members.” The second comprises “Educating Patients, Promoting Self-Management” and “Enlisting Help of Social and Community Support.” While maintaining the principle that implementing as many components While maintaining the principle that implementing as many components as possible is the first choice, when such an approach is not feasible, the results show that there are two different recipes with a low number of components sufficient for achieving the outcome.

### Economic evaluation

Twenty-one studies reported an economic analysis. Overall, the quality of the retrieved studies, as assessed through the Drummond 10-item scale [23] was medium to low (see Supplementary Table 3).

Four of these [31–34] carried out a cost-utility analysis or cost-benefit analysis, and 17 reported a cost-consequence analysis. The majority of the studies were conducted alongside randomized controlled trials or quasi-experimental studies. Only three studies undertook decision modeling analysis [31, 33, 35].

The majority of studies reported the expenditure and possible cost savings in a descriptive manner. The main goal of this kind of work was to monitor whether the component reduced hospital readmissions and consequently direct health-care costs, thereby demonstrating the net financial benefit of investing in TC programs. The cost measurements were not always accurately described (e.g., hours of nursing time, number of physician visits, lost workdays, gained life years), but only the aggregate were reported.

Fourteen out of 15 cost-consequences analyses found a positive impact on costs. Cost-consequences analysis studies aimed to show the net financial benefit of investing in TC programs. Therefore, they often compared the savings obtained thanks to TC by reducing readmission rates and emergency visits, that is, the reduction of the direct health-care costs in the component group versus that of the control group and then adding the program costs.

NFB = [(CG costs − TC costs) × *N* patients in TC program] − Program costs.

NFB = Net financial benefit.

TC costs = mean $ per Time (days, months) health-care direct costs in the component group.

CG costs = mean $ per Time (days, months) health-care direct costs in the control group.

If the TC program costs are equal or less then the savings obtained, it will be considered the right choice from an economic point of view. Even though not all the studies reported the program costs in the analysis, their inclusion is important to define the financial impact; for example, a TC program was probably effective in reducing the costs of hospital stays and emergency care attendance, but when the costs of the program were considered, the net gain in public health-care costs was not significant [48].

A full economic evaluation indicates that the implementation of a TC program is cost-effective or cost-benefiting [31, 32, 34]. Comparing two ways of managing TC programs: (nurse) home visits and (only) telephone calls. Both approaches aim to strengthen patient self-care ability and confidence through regular monitoring and education [31]. Home visits enable the care team to educate patients and caregivers more effectively on medication use and side effects. Home care provides face-to-face communication, resulting in more effectiveness, but it can be costly. Only one study provided a cost-effectiveness analysis of home visits and telephone calls and reported the differential benefits [31]. While both were cost-effective with respect to the control group, they showed that telephone calls are more cost-effective, particularly over a longer period (84 days vs. 28 days). Their results raise the hypothesis that in the short run (28 days), home visits can be the better option, whereas for longer periods (84 days) telephone calls are the most cost-effective strategy. The incremental cost-effectiveness ratio of call versus home was £3,538 per quality-adjusted life year gained at 84 days [31].

Where studies reported analysis on subpopulations according to risk strata, the cost reductions were concentrated in the patients at highest risk. One study found that TC was associated with a downward trend in costs in the 6 months following discharge in all but the lowest risk stratum, while the magnitude increased with clinical risk [36]. However, this cost difference reached statistical significance only for the highest risk (average monthly cost difference $970, chi-square = 14.94, *P* < 0.001) [34]. Synthesizing the evidence, a positive return on investment for TC is much more likely if intelligently targeted toward higher-risk patients (see appendix B).

## Discussion

Past and current policies and strategies [1–3] have focused increasingly on the components of the transition from hospital to home. This is a vulnerable period of discontinuity and an area of emerging costs [5–6]. The promotion of safe and efficient transitions of care is central to the reduction of readmission rates and associated costs and, ultimately to the improvement of the quality of patient care [11]. Therefore, it is important to reduce avoidable hospital admissions by implementing integrated discharge programmes that can ensure continuity of care after hospital discharge [4].

The present study has highlighted the importance of examining the role of organizational levers (system characteristics and TC organizational components) in facilitating creating a successful TC program. It also gathers key evidence on implementing TC programs’ economic impacts and benefits. In this light, the results of the systematic literature review are discussed in three areas: the role of TC components and their combinations in reducing readmissions, the evidence on system characteristics, and the evidence on economic benefits.

Previous literature has mainly analyzed individual organizational components of TC programs [13] and concluded that multiple elements are relevant for significant improvements in quality of care and reductions in readmission rates [56]. Our results show that (i) the ability to reduce readmissions is associated with the number of components included in the TC program, and (ii) not all components are equally important in influencing readmissions.

The results of this review confirm the association between the number of components and the ability to reduce readmission rates [16]. Studies with significant reductions in readmission rates had almost twice the number of components of other studies. The novelty of the present work is that the configuration analysis makes it possible to find the necessary conditions (components) and the combinations of components that, when implemented together, reduce readmissions. The analysis shows the existence of necessary conditions that need to be implemented in any TC program, namely “monitoring and management of symptoms after discharge” and “discharge planning”. They show that close clinical monitoring of a discharged patient for active symptoms is necessary to ensure an effective process. Discharge planning is also necessary, emphasizing the importance of identifying patient needs before discharge and implementing components before discharge.

These components alone are not sufficient; they need to be combined with other components to lead to an effective TC program in terms of readmission rates. There are two possible configurations. The first focuses specifically on the coordination of team members, and the other concentrates resources on patient education and engaging social and community support. Following the configuration approach, the two combinations can be seen as two alternative ways of achieving the same outcome. Identifying alternative ways of achieving good outcomes can be useful when decision-makers are faced with a lack of resources and cannot implement all 10 components.

Both combinations are a way of responding quickly to possible setbacks in health status. The first builds its responsiveness by allocating resources to improve communication and optimise the flow of information, such as medical records, between all professionals involved in the patient’s care. The second bases its responsiveness on empowering the patient and creating a home environment that is well-suited to supporting the patient’s care in order to limit relapses. The success of this combination may be due to the fact that patients have better self-advocacy regarding their condition and are better able to manage their condition at home.

System characteristics are underexplored in studies on TC interventions. As the results show, there was little attention to the system in the studies under review. None of the studies focused on the relationship between the system and outcomes in terms of size, type of structure, skill mix, and technology used to implement the TC program - elements that could be crucial to the program’s success. Not to mention that, unlike other interventions, in TC programs it is possible to have more than one system at a time (i.e. the hospital and the family or nursing home, etc.), potentially increasing the complexity and the importance of understanding the characteristics of these agencies that may influence TC effectiveness. In view of the relevance that system characteristics can have on TC effectiveness, this is an important gap in the literature that must be addressed in future studies. Some interesting evidence was found regarding the skill mix and the mode of coordination among professionals.

As outlined in the findings, four organizational models emerge the health-based monocentric, the social-based monocentric, the multidisciplinary team and the mono-specialist team. The monocentric model places emphasis on the health or social competence of one individual overseeing the transition. These models are clearly categorised into health-focused and social-focused models. The two polycentric models, characterised by shared responsibility for the transition care process among several professionals, focus on the team’s ability to manage complex situations, which may require either a diverse set of skills within the team or, conversely, the involvement of several professionals with similar skills to effectively meet the patient’s needs. The choice of model should, therefore be guided by considerations such as the type of patient and the nature of the system in which transitions take place.

The type of patient is intertwined with disease characteristics; for example, acute vs. chronic pathologies may influence model selection. Highly complex clinical situations may warrant a health-based monocentric model, while multidisciplinary clinician-led teams become relevant in cases involving multiple medical disciplines. The multidisciplinary team is beneficial in addressing the multidimensional needs of fragile populations across medical, social, psychological and functional domains. Conversely, a social monocentric model may be appropriate for patients whose care is closely linked to navigating the healthcare system, outpatient providers, social services and community resources. Patient characteristics, including socioeconomic status and low health literacy, may create environmental barriers or hinder the understanding of health information. In particular, a significant proportion of super-utilisers are of low socio-economic status, suggesting that the choice of model may be biased towards a social monocentric approach. In the case of complex diseases, the integration of social aspects with highly qualified medical professionals may lead to a multidisciplinary team. There is little or no information in the studies on the characteristics of the system in which the transition takes place. It may be important to consider not only the characteristics of the healthcare institution overseeing the discharge but also those of the institutions that care for the patient during the transition, such as the family, community hospitals or nursing homes. Ultimately, the model choice will depend on several factors, including the economic implications of the skill mix involved. This review highlights the need for further research to better identify the advantages and disadvantages of specific models in different settings.

From an economic perspective, TC programs are a potentially cost-saving and cost-effective strategy, but the evidence with a full economic evaluation is limited, and the quality of economic evaluations is generally low, so more research is needed. In addition, recent articles have shown a tendency to select patients for a TC program not according to specific pathology but according to their resource use - the so-called super-utilizers [55], who are higher-risk patients with multiple comorbidities, complex medical needs, and higher readmission rates. Although super-utilizers represent a small percentage of the total patient population, they often account for half of the nation’s total healthcare expenditure, so the absolute risk reduction and potential financial benefit of additional TC surveillance may be even more significant.

Despite the contributions made, the results need to be interpreted in the light of a number of limitations. The review was limited to studies published in English, which may introduce a language bias. Relevant studies published in other languages may have been overlooked. In conducting this systematic literature review, it is important to acknowledge the lack of direct involvement of patients, members of the public, or representatives of patient advocacy groups in the research process. Patient and public involvement (PPI) was not incorporated into the design, conduct or interpretation of this review. The study is a first attempt to assess the economic, organizational impact of the intervention, and the extensive engagement required for robust PPI was beyond the scope of the project. Future studies can incorporate PPI to improve the relevance, particularly of studies on system characteristics and organizational components of TC programs.

## Conclusions

Implementation of TC programs has received promising attention in terms of improving health outcomes the reduction of hospital unplanned readmission rates and costs, but systematic evidence on the organizational levers that can affect the success of these health programs is low [19]. The results of the present work indicate that much can be learned by focusing on organizational factors affecting the TC program results. Implementation of these complex programs requires several organizational choices, such as the types and the number of TC elements to be implemented, the professional skills to be involved, the types of patients to be involved, and the technological tools to be used. The complexity of TC programs can be studied using a configurational approach [21–22]. Further studies can use this approach to define different recipes for effective TC programs. This study gives preliminary insights to systematize organizational levers that can facilitate decision-makers’ choice of the most suitable model for their specific case.

### Electronic supplementary material

Below is the link to the electronic supplementary material.

Supplementary Material 1

## Data Availability

All data generated or analysed during this study are included in this published article [and its supplementary information files].
