# Correction: Disentangling organizational levers and economic benefits in transitional care programs: a systematic review and configurational analysis

**DOI:** 10.1186/s12913-024-10715-8

**Published:** 2024-02-27

**Authors:** Stefano Landi, Maria Martina Panella, Chiara Leardini

**Affiliations:** 1 Department of Management, Università di Verona, Via Cantarane, 24, 37129 Verona, Italy; 2 IRCCS- Azienda ospedaliera universitaria Bologna, Policlinico di S.Orsola-Malpighi, Via Pietro Albertoni, 15, Bologna, Italy

**Correction to: BMC Health Services Research (2024) 24:46**

10.1186/s12913-023-10461-3

In this article, the statement in the Funding section was incomplete. It was incorrectly given as ‘This research received no specific grant from any funding agency in the public, commercial, or not-for-profit sectors.’ and should have read ‘This research received no specific grant from any funding agency in the public, commercial, or not-for-profit sectors. The University of Verona is acknowledged for the support provided through the extraordinary university fund for Open Access publication.’

The original article has been corrected.

