# Supplementary material for: Disentangling organizational levers and economic benefits in transitional care programs: a systematic review and configurational analysis
Source: BMC Health Serv Res. 2024 Jan 9;24:46. doi: 10.1186/s12913-023-10461-3 (PMC10777542; doi:10.1186/s12913-023-10461-3)
Supplement: Supplementary file 1 — Supplementary Material 1 [file 12913_2023_10461_MOESM1_ESM.docx]

**Supplementary Materials**

**Supplementary table 1**: Main results of the study included in the systematic review.

| Author | Year | Title | Country | Methodology | Target population | Results |
| --- | --- | --- | --- | --- | --- | --- |
| Moore et al. | 2017 | Improving transition to postacute care for elderly patient using a novel video-conferencing program: ECHO-Care Transition | USA | Randomized controlled trial | Older patients | 30-day readmission rates are significantly lower in the intervention group, as are the 30-day total health-care costs and the average length of stay. |
| Hall et al. | 2014 | Cardiac surgery nurse practitioner home visit prevents coronary artery bypass graft readmission | USA | Retrospective observational cohort study | Cardiovascular patients | A home transition program providing continuity of care, significantly reduced the 30-day composite end point of readmission/death after CABG. |
| Wong et al. | 2012 | Cost-effectiveness of a health social partnership transitional program for post-discharge medical patients | China | Randomized controlled trial | Older patients | The readmission rates within 28 and 84 days are significantly higher in the control group. Utility values showed no difference between the control and study groups at baseline. The intervention had an 89% chance of being cost-effective at the threshold of £20,000/QALY. |
| Harrison et al. | 2014 | Assessing the impact of nurse post-discharge telephone calls on 30-day hospital readmission rates | USA | Retrospective observational study | General medical patients | In unadjusted analyses, patients who received a call and completed the intervention are significantly less likely to be readmitted compared with those who did not. |
| Wong et al. | 2015 | Economic evaluation of the differential benefits of home visits with telephone calls and telephone calls only in transitional discharge support | China | Randomized controlled trial | Chronic patients | The home arm is less costly but less effective at 28 days and is dominating (less costly and more effective) at 84 days. The call arm is dominant at both 28 and 84 days. The RCT showed that the bundled intervention involving home visits and calls was more effective than calls only in the reduction of hospital readmissions. |
| Stranges et al. | 2015 | A multidisciplinary intervention for reducing readmissions among older adults in a patient-centered medical home | USA | Retrospective cohort study | Older patients | When those completing the intervention are examined, readmission rates are significantly reduced. Likewise, time to readmission is significantly longer among those receiving the intervention and potential cost avoidance is observed only when the intervention is completed. |
| Anderson | 2005 | Benefits of comprehensive inpatient education and discharge planning combined with outpatient support in elderly patients with congestive heart failure | USA | Randomized controlled trial | Cardiovascular patients | The intervention resulted in a marked reduction in 6-month readmission rates. There is a registered average total cost-saving for each subject in the interventional group, based on the decreased utilization of both skilled nursing services and home health care during outpatient follow-up. |
| Courtney et al. | 2009 | Fewer emergency readmissions and better quality of life for older adults at risk of hospital readmission: a randomized controlled trial to determine the effectiveness of a 24-week exercise and telephone follow-up program | Australia | Randomized controlled trial | Older patients | The intervention group used significantly fewer emergency health services after discharge, an outcome with considerable benefits for patients and health service providers. The results from this sample indicate absolute reductions of 20% in hospital readmissions and 40% in emergency use of local GP medical services. |
| Galbraith et al. | 2017 | Long-term impact of a post-discharge community health worker intervention on health care costs in a safety-net system | USA | Randomized controlled trial | Chronic patients | Total costs per patient over the 180 days post-index discharge for those aged ≥ 60 years were significantly lower for PN patients. |
| Jackson et al. | 2013 | Transitional care cut hospital readmissions for North Carolina Medicaid patients with complex chronic conditions | USA | Observational study | Chronic patients (with a risk readmission profile) | They found that those who received transitional care are 20% less likely to experience a readmission during the subsequent year than clinically similar patients who received usual care. Benefits of the intervention are greatest among patients with the highest readmission risk. One readmission is averted for every six patients who received transitional care services and one for every three of the highest risk patients. |
| Watkins et al. | 2012 | Hospital to home: a transition program for frail older adults | USA | Randomized controlled trial | Older patients (with a risk readmission profile) | Hospital readmissions are decreased by 61% for this high-risk population. Cost savings are registered by preventing readmissions. |
| Stauffer et al. | 2011 | Effectiveness and cost of a transitional care program for heart failure | USA | Randomized controlled trial | Older cardiovascular patients | The intervention significantly reduced adjusted 30-day readmission by 48% during the post-intervention period. The intervention had little effect on length of stay or total 60-day direct costs. Under the current payment system, the intervention reduced the hospital financial contribution margin. |
| Naylor et al. | 2004 | Transitional care of older adults hospitalized with heart failure: a randomized, controlled trial | USA | Randomized controlled trial | Older cardiovascular patients | Time to first readmission or death was longer in intervention patients. At 52 weeks, intervention group patients had fewer readmissions and lower mean total costs. |
| Jingyi et al. | 2018 | Cost-benefit analysis of transitional care in neurosurgery | USA | Observational study | Neurosurgery patients | The neurosurgical TCP is associated with decreased costs of initial admissions, 30-day readmissions, and total costs of hospitalization alongside previously published decreased length of stay and reduced 30-day readmission rates. |
| Jackson et al. | 2016 | Incremental benefit of a home visit following discharge for patients with multiple chronic conditions receiving transitional care | USA | Randomized controlled trial | Chronic patients | In multivariate analysis, home visits significantly reduced the odds of readmission within 30 days. At the 6-month follow-up, home visits were associated with fewer inpatient admissions within 4 of 6 clinical risk strata, and lower total costs of care for highest risk patients. For complex chronic patients, home visits reduced the likelihood of a 30-day readmission by almost half compared to less intensive forms of nurse-led transitional care support. Higher risk patients experienced the greatest benefit in terms of number of inpatient admissions and total cost of care in the 6 months following discharge. |
| Russel et al. | 2011 | Implementing a transitional care program for high-risk heart failure patients: findings from a community-based partnership between a certified home healthcare agency and regional hospital | USA | Retrospective observational study | Older cardiovascular patients (with a risk readmission profile) | Analyses indicated that patients who received the transitional care services were significantly less likely to be readmitted to the hospital than the patients in the control group. |
| Saleh et al. | 2012 | An effectiveness and cost-benefit analysis of a hospital-based discharge transition program for elderly Medicare recipients | USA | Randomized controlled trial | Older patients | The 1-year readmission analysis revealed that control participants are more likely to be readmitted than intervention participants. Findings from the cost-benefit analysis revealed a favorable cost-benefit. |
| Kam et al. | 2015 | Effects of a 4-week transitional care programme for discharged stroke survivors in Hong Kong: a randomised controlled trial | China | Randomized controlled trial | Cardiovascular patients | The intervention group had lower hospital readmission and use of emergency room rates, but only the use of emergency room had a significant difference when compared with control. |
| Zhang et al. | 2017 | Effects of a nurse-led transitional care programme on readmission, self-efficacy to implement health-promoting behaviours, functional status and life quality among Chinese patients with coronary artery disease: a randomized controlled trial | China | Randomized controlled trial | Cardiovascular patients | The difference in readmission rates and physical limitations was not significant between the two groups. |
| Dhillon et al. | 2017 | Impact of “transition of care model” on hospital diabetic ketoacidosis readmission rates: a pilot study | USA | Retrospective chart review study | Diabetic adults (>18) with ketoacidosis (DKA) | Contacting patients within a few days after discharge was an effective way to reinforce the discharge plan, clarify misunderstandings, accomplish early detection of unexpected outcomes, and remind patients about outpatient follow-up. The study could not show a statistically significant reduction in hospital readmission rates, but found a trend towards increased outpatient follow-up. |
| Simpson | 2014 | A quality improvement plan to reduce 30-day readmissions of heart failure patients | USA | Evidence-based quality initiative | Older cardiovascular patients | The 30-day readmission rate for HF patients for the 5-month period of the project was 18.95%—a decrease of 8.5% |
| Coleman et al. | 2006 | The care transitions intervention results of a randomized controlled trial | USA | Randomized controlled trial | Chronic older patients | Intervention patients had lower rehospitalization rates at 30 days and at 90 days than control subjects. Intervention patients had lower rehospitalization rates for the same condition that precipitated the index hospitalization at 90 days and at 180 days than controls. The mean hospital costs were lower for intervention patients vs controls at 180 days. |
| Naylor et al. | 1999 | Comprehensive discharge planning and home follow up of hospitalized elders | USA | Randomized controlled trial | Older patients | An advanced practice nurse-centered discharge planning and home care intervention for at-risk hospitalized elders reduced readmission, lengthened the time between discharge and readmission, and decreased the cost of providing health care. |
| Naylor et al. | 1994 | Comprehensive discharge planning for the hospitalized elderly | USA | Randomized clinical trial | Older patients | Patients in the medical intervention group had fewer readmissions, fewer total days rehospitalized, lower readmission charges, and lower charges for health-care services after discharge. |
| Rich et al. | 1995 | A multidisciplinary intervention to prevent the readmission of elderly patients with congestive heart failure | USA | Prospective randomized trial | Older cardiovascular patients | A nurse-directed, multidisciplinary intervention can improve quality of life and reduce hospital use and medical costs for elderly patients with congestive HF. |
| Balaban et al. | 2015 | A patient navigator intervention to reduce hospital readmissions among high-risk safety-net patients: a randomized controlled trial | USA | Randomized clinical trial | General medical patients | 30-day readmission rates did not differ between intervention and control patients. However, the two age groups demonstrated marked differences. Patient navigator intervention among high risk, safety-net patients decreased readmission among older patients while increasing readmissions among younger patients. |
| Graves et al. | 2009 | Cost-effectiveness of an intervention to reduce emergency re-admissions to hospital among older patients | Australia | Randomized clinical trial | Older patients | The intervention was a comprehensive nursing and physiotherapy assessment and an individually tailored program of exercise strategies and nurse home visits with telephone follow-up, commencing in hospital and continuing following discharge for 24 weeks. The change to cost outcomes, including the costs of implementing the intervention and all subsequent use of health-care services, and, the change to health benefits, represented by QALY, were estimated for the intervention as compared to existing practice. |
| Kwok et al. | 2008 | A randomized controlled trial of a community nurse-supported hospital discharge programme in older patients with chronic heart failure | China | Randomized clinical trial | Older cardiovascular patients | Community nurse-supported post-discharge programme was effective in preserving independence and was probably effective in reducing the number of unplanned readmissions. The cost benefits to public health care were not significant. |
| Wong et al. | 2008 | Can home visits help reduce hospital readmissions? Randomized controlled trial | China | Randomized clinical trial | Chronic patients | Preventive home visits were not effective in reducing hospital readmissions, but satisfaction with care was enhanced. |
| Gardner et al. | 2014 | Is implementation of the care transitions intervention associated with cost avoidance after hospital discharge? | USA | Quasi-experimental cohort study | General medical patients | The intervention group had significantly lower utilization in the 6 months after discharge and lower mean total health-care costs. |
| Lee | 2017 | Transitional care intervention: a readmission solution | USA | Quasi-experimental design with a retrospective group | General medical patients | Results indicated a significant difference in the 30-day readmission rate among cardiac transplant patients, with a larger percentage encountered in the usual care group. The transitional care intervention strategy resulted in statistically significant fewer 30-day readmissions as compared with usual care. |
| Baley et al. | 2019 | Effect of intensive interdisciplinary transitional care for high-need, high-cost patients on quality, outcomes, and costs: a quasi-experimental study | USA | Quasi-experimental study | General medical patients (identified as medically underserved and readmission hotspots ≥ 2 inpatient admissions or one inpatient admission and ≥ 2 ED visits in the 6 months) | Care transition models emphasizing strong interdisciplinary patient engagement and rapid primary care follow-up can enable health systems to improve quality and outcomes while reducing costs among high-need, high-cost Medicaid patients. |
| Candelario et al. | 2018 | Impact of a centralized interdisciplinary discharge unit on readmission rates and transitional care services in high risk patients | USA | Retrospective chart review | Chronic patients (with a risk readmission profile) | Interdisciplinary transitions of care unit dedicated to discharge services was associated with a reduction in all-cause pneumonia hospital readmission rates and ED visits when compared with standard hospital discharge. |
| Kripalani et al. | 2019 | A transition care coordinator model reduces hospital readmissions and costs | USA | Quasi-experimental evaluation design | Chronic patients | An evidence-based multi-component intervention delivered by nurse TCCs reduced 30- and 90-day readmissions and associated health-care costs. |
| Lee et al. | 2019 | Outcomes of a transitional care clinic to reduce heart failure readmissions at an urban academic medical center | USA | Randomized clinical trial | Cardiovascular patients | Among high readmission risk and predominantly Black population with HF, TCC resulted in significantly lower hospital readmission rates within 7 days and within 30 days of initial discharge. |
| Rahim et al. | 2018 | The role of continuous care model on hospital readmission of patients with heart failure: a randomized controlled clinical trial | Iran | Randomized clinical trial | Cardiovascular patients | The results confirmed that the application of the intervention could significantly reduce the readmission rates in HF patients and thus lead to decreased care costs and increased the quality of life in patients with HF. |
| Taylor et al. | 2019 | A population health approach to transitional care management for high-risk patients with diabetes: outcomes at a rural hospital | USA | Observational study | General medical patients (with a risk readmission profile) | The post-intervention 30-day readmission rate was 18.0% among patients identified as high or very high risk versus 8.8% among the overall population and did not differ significantly between TCM participants with diabetes and those without. |
| Xiang et al. | 2018 | Social work-based transitional care intervention for super utilizers of medical care: a retrospective analysis of the bridge model for super utilizers | USA | Retrospective, single group, pre-post design | Super-utilizers with ≥ 5 inpatient admissions in a 12-month period | The analyses revealed significant reductions in the total number of hospital admissions, 30-day readmission rates, number of ED visits, average hospital charges per episode, and total hospital charges per person after the intervention. |

Abbreviations: CABG, coronary artery bypass graft; QALY, quality-adjusted life year; RCT, randomized controlled trial; GP, general practitioner; PN, patient navigator; BMCG, Baylor Medical Center Garland; TCP, transitional care program; DKA, diabetic ketoacidosis; HF, heart failure; ED, emergency department; TCC, transition care coordinator; TCM, transitional care management.

**Supplementary image 1:** Trend of publication of articles over time in total

Supplementary table 2: Qualitative comparative analysis. Configurations of interventions leading to an effective TC program in terms of readmission at 30 and 60 days

| Configurations | 1 | 2 |
| --- | --- | --- |
| Complete Communication of Information |  |  |
| Availability, Timeliness, Clarity, and Organization of Information |  |  |
| Medication Safety |  |  |
| Educating Patients, Promoting Self-Management |  | ● |
| Monitoring and Managing Symptoms after Discharge | ● | ● |
| Enlisting Help of Social and Community Supports |  | ● |
| Advance Care Planning |  |  |
| Coordinating Care Among Team Members | ● |  |
| Discharge Planning | ● | ● |
| Follow-up with Outpatient Providers |  |  |
| Consistency | 0.96 | 0.98 |
| Raw coverage | 0.74 | 0.35 |
| Unique coverage | 0.08 | 0.02 |
| Number of cases | 36 | 36 |
| Solution coverage | 0.78 |  |
| Solution consistency | 0.96 |  |

Following the guidelines suggested by Ragin and Fiss (2008), we graphically report the results to highlight the presence and absence of the variables in each combination as shown in the table, where black circles (●) indicate the presence of a condition and circles with an x (⊗) indicate its absence. Further, a blank cell indicates the do not care condition, which means that the antecedent is not relevant in that configuration. As shown, none of the antecedents alone can sufficiently explain a high intention to stay among residents. This finding shows that no single antecedent alone leads to the outcome and must be combined with at least some others.

**Supplementary table 3:** Summary of the main economic characteristics of the studies reporting the quality of the study, the adopted perspective, the time horizon chosen, the types of costs included in the analysis and the main results.

|  |  | Drummond  scale |  | Perspective | Time horizon | Costs included | Main results |
| --- | --- | --- | --- | --- | --- | --- | --- |
| 1 | Moore et al. 2017 | 2 | CCA | Hospital | 30 days | Direct health-care costs | Reduced hospital readmission rates, and total health-care spending within 30 days |
| 2 | Hall et al., 2014 | 3 | CCA | Hospital | 30 days | Direct health-care costs  +  Cost of the nurse practitioner | Because the control group had 12 more readmissions than the TC group, TC program led to €262 in cost-saving per patient |
| 3 | Wong et al. 2012 | 8 | CUA | Health system /third payer | 28 days and 84 days | Direct health-care costs  +  Program costs (micro costing) | The intervention had an 89% chance of being cost-effective at the threshold of £20,000/QALY |
| 4 | Wong et al., 2015 | 8 | CUA | Societal—Health system | 28 days and 84 days | Direct health-care costs  +  Program costs  +  Pre-intervention costs | TC dominated at both 28 and 84 days. The bundled intervention involving home visits and calls was more effective than calls only in the reduction of hospital readmissions at 28 days, but calls only have a higher chance of being cost-effective for a sustained period after intervention |
| 5 | Anderson et al., 2005 | 6 | CCA | Hospital | 6 weeks | Direct health-care costs +  Home care visit utilization | TC group had less use of resources and fewer visits, leading to cost savings |
| 6 | Galbraith et al, 2017 | 6 | CCA | Hospital / Health system | 180 days | Direct health-care costs  +  Program costs (Nurse salary) | TC lowered the costs for patients aged ≥60 years while increasing costing for high-risk patients under age 60 |
| 7 | Stauffer et al.2011 | 4 | CCA | Hospital | 60 days | Direct health-care costs  +  Program costs | The intervention had little effect on length of stay or total 60-day direct costs |
| 8 | Naylor et al. 2004 | 6 | CCA | Societal | 52 weeks | Direct health-care costs  +  Program costs (nurse and materials) | The net financial benefit of the higher costs of the program was positive thanks to the reduction in acute visits, ED, and hospitalizations |
| 9 | Jingyi et al 2018 | 6 | CCA | Hospital | 30 days | Direct health-care costs  +  Program costs (Nurse salary) | The TCP was associated with an average total cost reduction of 17.2% owing to decreased length of stay and reduced 30-day readmission rates |
| 10 | Jackson et al. 2016 | 4 | CCA | Third payer | 30 days, 180 days | Direct health-care costs | Cost savings for highest risk patients owing to lower readmissions |
| 11 | Saleh et al, 2012 | 6 | CBA | Hospital | 30 days, 90 days  365 days | Direct health-care costs  +  Program costs | Cost-benefit ratio of 1.09 |
| 12 | Coleman et al, 2006 | 6 | CCA | Hospital | 90 days  180 days | Direct health-care costs  +  Program cost | Positive net financial benefit from lower readmissions. |
| 13 | Naylor et al., 1999 | 5 | CCA | Third payer and patient | 24 weeks | Direct health-care costs | Reduced hospital readmission rates and total health-care spending |
| 14 | Naylor et al., 1994 | 4 | CCA | Third payer | 2, 6, 12 weeks | Direct health-care costs | Lower readmission charges and lower charges for health-care services after discharge |
| 15 | Rich et al, 1995 | 6 | CCA | Third payer and Societal | 90 days | Direct health-care costs  +  Program cost + indirect costs | Because of the reduction in hospital admissions, the overall cost of care was $460 less per patient in the treatment group. |
| 16 | Graves et al., 2009 | 8 | CUA | Hospital | 24 weeks | Direct health-care costs  +  Program cost | The mean net-monetary-benefit per individual for the intervention group compared with the usual care condition was $7,907 |
| 17 | Kwok et al., 2008 | 6 | CCA | Third payer and societal | 6 months | Direct health-care costs  +  Program cost + Direct non-health-care costs | No statistically significant differences |
| 18 | Gardner et al., 2014 | 6 | CCA | Third payer | 6 months | Direct health-care costs | Positive net financial benefit from lower readmissions |
| 19 | Baylor et al., 2019 | 5 | CCA | Third payer | 6 months | Direct health-care costs | Fewer readmissions and reduced medical expenditures |
| 20 | Kripalani et al., 2019 | 5 | CCA | Hospital | 30, 90 days | Direct health-care costs  +  Program cost | Positive net financial benefit from lower readmissions |
| 21 | Xiang et al., 2019 | 4 | CCA | Third payer | 12 months | Direct health-care costs | Fewer readmissions and reduced medical expenditures |

Abbreviations: ED, emergency department; CCA, cost-consequences analysis; CUA, cost-utility analysis; CEA, cost-effectiveness analysis; CBE, cost-benefit analysis; QALY, quality-adjusted life year; TC, transitional care; TCP, transitional care program.
